# Why and Where to Fund Carbon Capture and Storage

**DOI:** 10.1007/s11948-021-00344-3

**Published:** 2021-11-18

**Authors:** Kian Mintz-Woo, Joe Lane

**Affiliations:** 1 Department of Philosophy and Environmental Research Institute, University College Cork, 4 Elderwood, Cork, Ireland; 2 Equity and Justice Research Group, International Institute for Applied Systems Analysis, Laxenburg, Austria; 3 University Center for Human Values, High Meadows Environmental Institute, Princeton School for Public and International Affairs, Princeton University, Princeton, NJ USA; 4 Andlinger Center for Energy and Environment, Princeton Institute for International and Regional Studies, Princeton University, Princeton, NJ USA

**Keywords:** Carbon capture and storage, Climate change, Climate justice, Global distributive justice, Legitimate expectations, Paris agreement

## Abstract

This paper puts forward two claims about funding carbon capture and storage. The first claim is that there are moral justifications supporting strategic investment into CO_2_ storage from global and regional perspectives. One argument draws on the empirical evidence which suggests carbon capture and storage would play a significant role in a portfolio of global solutions to climate change; the other draws on Rawls’ notion of legitimate expectations and Moellendorf’s Anti-Poverty principle. The second claim is that where to pursue this strategic investment poses a morally non-trivial problem, with considerations like near-term global distributive justice and undermining legitimate expectations favouring investing in developing regions, especially in Asia, and considerations like long-term climate impacts and best uses of resources favouring investing in the relatively wealthy regions that have the best prospects for successful storage development.

## Introduction

Despite three decades of growing intention to avoid dangerous climate change, progress in decarbonising the global economy has been insufficient to address growing emissions of carbon dioxide (CO_2_) from fossil fuel use. Furthermore, while the 2016 Paris Agreement (PA) achieved a historic international commitment to constrain global temperature rise to well below 2 °C, the plans put in place since then leave us well short of the trajectory needed to meet that goal (Minx et al., 2018). The result is a growing sense of urgency in the search to identify and implement abatement solutions for ongoing and historical CO_2_ emissions. That in turn leads to the consideration of more radical measures, accepting that we also have less time to wait for mitigation technologies to mature. These options cannot and should not supplant other mitigation measures, but there is evidence that they may be necessary additions to a portfolio of climate actions.

In this paper, we specifically focus on the technological option of Carbon Capture and Storage (CCS), a process which involves the injection of CO_2_ into underground geological features for sequestration over the long-term. This covers both the capture of large scale, point-source CO_2_ emissions resulting from the use of *fossil fuels*^1^ (FF-CCS), and the *carbon dioxide removal* process of capturing and sequestering CO_2_ that is already in the atmosphere (CDR-CCS).^2^

While industrial scale CCS facilities have been operating successfully for 25 years (Greig et al., 2016), uptake of the technology has repeatedly failed to deliver on hopes for a transformative infrastructure rollout (Arranz, 2015; Backstrand et al., 2011; IEA, 2018). Despite that, those hopes remain entrenched in the most recent wave of global scenarios for meeting the PA goal, with the modelled transitions strongly reliant on the implementation of CCS at massive scales. Notably, the CCS sequestration in those scenarios tends to be spread evenly across both FF-CCS vs CDR, with deployment concentrated more heavily in the developing world (e.g., 30–45% in developing Asia) than in those OECD regions with the majority of actual CCS experience (Lane et al., 2021).

This paper puts forward two claims on the topic of CCS that we view as being independent—i.e. one can accept either without accepting both.

First, we provide two lines of argument to claim that, despite valid moral objections to the risks of doing so, CCS should be pursued at large scale through strategic investment into CO_2_ storage assessment.

Second, we draw attention to the practically important question of *where* regionally we would want that initial stimulus funding to be aimed. While previously unrecognised by philosophers, we claim this question is both morally important and morally non-trivial. Specifically, it is subject to competing moral considerations, some providing a case for directing CCS stimulus funding to developing Asian regions, while others favour wealthier regions with the best prospects for successful CCS implementation.

We conclude by posing questions for further research.

## Why Pursue Carbon Capture and Storage

To build our case for pursuing large-scale CCS, we first outline which of the multiple practical and moral objections are most valid, then respond that CCS can be viewed as both a *Necessary Global Solution* and a *Justifiable Regional Solution* for meeting the PA objective. We then show that several of the moral objections discussed in the literature can be alleviated by endorsing early, strategic commitments to pursuing CCS development.

### Objections to CCS

The literature on the ethics of CCS is relatively small, primarily centred on a number of objections we categorise here as being concerned with (1) the risks directly associated with CCS operational activities; and (2) the planning risks inherent in an over-reliance on large-scale, future deployment of CCS. Much of that criticism is embedded in broader objections to the concepts of CDR and geo-engineering, with which CCS is often lumped.^3^

#### Risks Associated with CCS Operations

The most prominent CCS operational risks are associated with the underground injection of CO_2_, including the potential for increased local seismicity, groundwater contamination, and need for secure long-term containment. A prevailing view amongst technical experts is that, because of the extensive operational and modelling experience available, careful site selection can minimise these risks to a level viewed as comparable with other socially acceptable industrial activities (Celia, 2017; Meadowcroft & Langhelle, 2009). Regardless, the potential for these geophysical risks, along with other localised socio-economic concerns (e.g. the potential impact on real estate values) were prominent in the community objections that overwhelmed Dutch and German attempts to instigate industrial scale CCS (Shackley & Dütschke, 2012).^4^

That strong understanding of geological CO_2_ injection processes means that CCS would carry considerably fewer systemic or unknown risks associated with learning-by-doing than would the geoengineering techniques with which CCS sometimes gets bundled in the literature. For the much more speculative geoengineering techniques, such as solar radiation management (Flegal et al., 2019) and iron fertilisation of oceans (Hale & Dilling, 2011), even research and small scale testing can risk substantial spillovers into regional and international environments (Morrow et al., 2009; Preston, 2013).

Discussions of CCS also get conflated with the broader objection that large-scale CDR techniques risk major disruption to other socio-economic and ecological systems, such as the potential for large-scale BECCS to divert land and water away from food production (Anderson & Peters, 2016; Fuss et al., 2014; Minx et al., 2018; Preston, 2013; Shue, 2017). We do not challenge those concerns over nutrition and other morally weighty goals (although cf. Callies & Moellendorf, 2021 for a rebuttal); however, note that they don’t of themselves undermine the case for pursuing large-scale CCS deployment (Lenzi, 2021). The issues are whether these risks can be alleviated and whether they are outweighed by climate risks. Although we do not have the space to outline this fully here, we believe that some of these risks can be lessened. Some also argue that the climate risks are greater than the BECCS risks (cf. Callies & Moellendorf, 2021; Peacock, 2021), especially when it is recognised that climate change will *itself* exacerbate land and resource constraints. However, this highlights the dilemma about choice of storage development discussed below, where local costs and benefits might trade off against global mitigation capacity.

#### Risks Associated with CCS Expectations

The second category of literature objections relates to the long-running criticism that CCS-based solutions are *unproven at the scale needed* to make major contributions to the deep economic decarbonisation required for meeting the PA goals. This charge was led by environmental NGOs in earlier debates on FF-CCS (Corry & Riesch, 2012), with researchers recognising that the question of scalability was not just technical, but intrinsically tied to our capacity for regulatory and socio-political transition (Backstrand et al., 2011). More recently, many have raised this concern with respect to BECCS specifically and CDR more generally (Lenzi, 2018; Shue, 2017), albeit with a primary focus on constraints (e.g. biomass production) not directly associated with CCS. It is often assumed that CO_2_ storage potential is unlikely to be a serious challenge (Fuss et al., 2014; Minx et al., 2018).

Regarding CCS specifically, a recent critique (Lane et al., 2021) suggests this concern about a potential storage bottleneck might be even more important than appreciated in both technical and moral research communities. The problem is not that the CCS process is unproven per se—CCS is considered to be at a high level of technological readiness (Bui et al., 2018), with our understanding of underground storage operations (Harding et al., 2018; Ringrose, 2018) and risk (Celia, 2017) inspiring confidence that CCS at large industrial- and regional-scales is a realistic proposition. However, the uncertainties inherent to storage evaluation mean that the practicable *size* of the ultimate storage opportunity and the practicable *rate* at which we could expand geological storage infrastructure both remain unproven. We therefore believe it unwise to accept assertions that geological CO_2_ storage won’t be a constraint on meeting the time-frame based CCS deployment set out in PA-compliant scenarios.

Those scalability doubts underlie the prominent *moral hazard* critique that expectations of a successful CDR-CCS (Anderson & Peters, 2016; Gardiner, 2014; Lenzi, 2018; Lenzi et al., 2018; Morrow, 2014) or FF-CCS (Backstrand et al., 2011; McLaren, 2012; Stephens, 2014) deployment would lead policymakers to fail in pursuing other mitigation options as ambitiously as they otherwise might. An extension of those concerns is that decision makers might be encouraged to accept a short-term *overshoot* of the global CO_2_ emissions budget, assuming that we can subsequently use CDR to draw back down the excess atmospheric CO_2_ and still meet targets for constraining global temperature increase (Meadowcroft, 2013). For Shue (2017, 2018), such a “climate recovery strategy” would represent an extraordinary and unjustified case of intertemporal risk transfer, given the possibility that irreversible impact thresholds, or tipping points, might be crossed before the CDR drawdown has succeeded (also cf. Lenton, 2018).

With a more general eye to the likelihood of failure, Lenzi (2018) connects the scale of expectations with the scale of possible externality risks, warning of *hubris* in relying on humankind’s ability to overcome these challenges through large-scale technological innovation and implementation (see also Preston, 2013). A decade earlier, Meadowcroft and Langhelle (2009) identified the impossibility of perfect foresight about the risks of a global-scale implementation of CO_2_ storage.

Distinguishing CCS from broader CDR and geoengineering concerns again helps; however, we draw nuanced conclusions. The objection to relying on a CO_2_ emissions overshoot, while compelling, relates specifically to CDR and doesn’t obviate the need to consider ambitions for CCS to mitigate fossil fuel emission sources. On the other hand, the more general concerns of moral hazard and hubris retain their force, given that the technical research community may have overestimated the practicable potential for CCS deployment within the timeframes necessary for PA compliance.

### Moral Arguments in Favour of CCS

Despite the salience of the risks caused by unrealistic expectations, we do not view them as a reason to reject the option of CCS. Here, we outline our contrary position that there are two arguments supporting the pursuit of CCS. The first, drawing on modelling results, argues that CCS plays a major role in any feasible portfolio of climate responses to PA targets. The second, drawing on moral claims about protecting economic and energy access, argues that CCS capacity should be available to regions with hard to decarbonise infrastructure.

Both arguments begin by accepting that a moral imperative exists to mitigate CO_2_ emissions sufficiently to meet the ‘well below 2 °C’ target of the PA, because of a strong moral duty to avoid the grave harms caused by climate change above that level (IPCC, 2018). Stern (2014a, b) outlines the variety of views that converge on this as being morally required.

#### A Necessary Global Solution

Our first argument takes the evidence from energy and environment models to suggest that, at the very least, very significant levels of CCS are part of the portfolio of climate actions necessary for PA-compliance. Firstly, integrated assessment models provide their lowest cost version of an energy-economy transition, subject to constraints on mitigation options. As a result, we can deduce that meeting this goal without one of the most important options would impose either a net increase in cost on the overall solution, a net increase in the time taken to decarbonise, or likely both. In other words, removing CCS as an option would impose some mix of higher cost, higher risk, and slower decarbonisation, making it less likely that the PA goal would be met (Morrow et al., 2018).

The complex implications of constraining CCS are illustrated by the International Energy Agency (IEA, 2019a), whose modelling indicates that constraining the FF-CCS contribution would increase transition costs, substantially increase the need for electricity generation, and increase reliance on alternatives at an lower level of technological readiness. Studies that severely curtail all CCS deployment (Gambhir et al., 2017), or eliminate it completely (Riahi et al., 2015), show increases in long term transition costs ranging from 45 to 100%. Arguably more important is the repeated finding that, without CCS included as an option, the probability that the modelled outcomes are consistent with PA targets diminishes dramatically, sometimes to the point of infeasibility (Luderer et al., 2018; Riahi et al., 2015; Rogelj et al., 2013).

A second perspective provided by the integrated assessment model results is that, to the extent they can be taken to imply the limits of transition feasibility, we may quickly be approaching a point where the PA goal might be unattainable without a strong reliance on CDR-CCS. Reviews of the modelling literature (Hilaire et al., 2019; Minx et al., 2018) indicate that a temperature increase limit of 1.5 °C already appears impossible without a large CDR rollout; and that meeting a 2 °C target without large-scale CDR would need an extraordinary ramp up of mitigation activity by the year 2030 that far exceeds what has been promised under the national commitments following the Paris Agreement.

Recognising the many uncertainties involved, Minx et al. (2018) put forward the compelling argument that we must pursue multiple types of CDR if their aggregate portfolio is to be ramped up to the required scale. Most importantly, assessments of global CDR prospects (Hepburn et al., 2019; Minx et al., 2018; Turner et al., 2018) suggest that the scale of modelled CDR would seem highly improbable without major contributions from both land-use change and CCS. Given others have already suggested those same rates of required land-use change seem unrealistic (Brown et al., 2019; Turner et al., 2018), we can emphasise the burden of expectation placed on CCS.

Recognising the strong dependence on CCS in those model results, we construct our *Necessary Global Solution* Argument as follows:

1. It is a moral imperative to achieve the PA goal
2. If there is a moral imperative to achieve the PA goal, prudential grounds require that we should pursue all necessary means to that end
3. One of those necessary means is to pursue large-scale CCS (at least CDR-CCS)
4. Therefore, we should pursue large-scale CCS (from 1, 2, 3)

For premise 3, we draw on the two perspectives above, so as to underline the importance of CCS to global decarbonisation.

If premises 1–3 hold, then conclusion 4 follows; to meet our moral obligations, we should pursue CDR-CCS sufficient to meet the goal of the Paris Agreement.^5^

#### A Justifiable Regional Solution

Our second argument for pursuing CCS draws on two ethical principles to argue that CCS is a *Justifiable Regional Solution*:

1. It is a moral imperative to reach the PA goal
2. If there is a moral imperative to achieve the PA goal, prudential grounds require that we should pursue all necessary means to that end
3. We cannot reach the PA goal without it being imperative for all major regions to achieve significant mitigation
4. Therefore, all major regions should achieve significant mitigation (from 1, 2, 3)
5. If certain regions do not have large-scale access to CCS, achieving significant mitigation would disrupt their economic and energy systems
6. Therefore, without large-scale access to CCS, such regions could not meet their mitigation obligations without disrupting their economic and energy systems (from 4, 5)
7. Citizens require predictable economic and energy systems in order to pursue their conceptions of the good life, i.e. they have legitimate expectations to predictable economic and energy systems
8. The need for citizens to pursue conceptions of the good life comes with a defeasible moral obligation to avoid conditions which undermine legitimate expectations, i.e. there is a defeasible moral obligation to avoid disruption to economic and energy systems
9. We have a moral obligation to support developing regions to access those mitigation options that least imperil their development
10. Therefore, in certain regions, there is a (defeasible) moral obligation to pursue large-scale CCS (from 6, 7, 8 or 6, 9)

Premise 3 follows a similar empirical logic to premise 3 of the *Necessary Global Solution* (§2.2.1), this time with a focus on the regional spread of mitigation in PA-compliant scenarios.

Again, we infer that failing to deliver on any one of the larger mitigation contributions would significantly increase climate risks. Along with the most CO_2_ intensive large OECD economies, PA-compliant transition scenarios consistently rely on the largest developing economies to make major mitigation contributions.

Premise 5 recognises that many regions’ energy systems rely heavily on fossil fuelled energy to provide multiple socio-economic services that are challenging and/or costly to replace with low-CO_2_ options. First, utilising domestic fossil fuel supply provides a cheap energy source that enhances domestic energy security, and supports local supply chains (e.g. mining, transport) that deliver benefits across broad segments of society. Fossil fuelled electricity generation also provides a stabilising role for electricity grids, necessary to support growing output from variable renewable electricity generation (Sepulveda et al., 2018). Furthermore, many localised economies depend strongly on existing materials production (cement, steel, fertiliser, chemicals) industries that are heavily reliant on fossil fuel inputs, but with limited technological alternatives for mitigation.

Where those concerns hold, the implementation of FF-CCS would allow the partial continuation of those valuable socio-economic services, thereby reducing the disruption that mitigation would impose on the domestic energy economy.

For developing countries, PA-compliant mitigation could also require that many of the materials and electricity production facilities be decommissioned well before their economic lifetime is reached. Any enforced closure of large industrial facilities is likely to disrupt socio-economic conditions in the surrounding locale. Premature retirement creates the additional economic stress associated with the lost return on sunk capital.

In support of premise 7, while conceptions of the good may vary in their requirements for energy, Meyer and Sanklecha (2011) make a convincing case that many if not most legitimate expectations require predictability. The key idea is that, if the governments are substantively just and the citizens have accepted the rules of those governments as just, there arises legitimate expectations that those governments will function similarly and deliver benefits in accordance with that scheme. In this case, where the relevant expectations concern energy and the economy, citizens legitimately develop expectations that development and energy provision will be there to facilitate pursuit of the good life. This is pertinent for regional economic systems, given economic shocks can be highly damaging to the lives of populations. A similar case can be made for the energy-economy more specifically.

Premise 8 is a moral claim drawn from Rawls (1999, p. 207), who was concerned about these legitimate expectations. The moral claim is that, insofar as that helps in their pursuit of the good life, there is a strong, but defeasible, obligation to meet these legitimate expectations (Meyer & Sanklecha, 2011, 2014).

Premise 9 draws on the ‘Antipoverty Principle’ of Moellendorf (2014), which holds that the preferred mitigation options are those that minimise the mitigation costs imposed on developing regions, and least imperil their development.

Beginning from premise 6, both the first (premises 7 and 8) and second (premise 9) moral routes support our final conclusion 10: that there is a (defeasible) moral obligation to pursue large-scale CCS in certain regions. The first route says that legitimate expectations—not just in terms of development—should not be undercut by governments. The second, that large-scale CCS is justified as the mitigation option for some CO_2_ sources that imposes the least cost and disruption to the development pathway in developing regions. For those developing regions, we take the argument’s conclusions to be more robust given that there is more than one way to justify them.

### The Case for Committing to a Strategic Pursuit of CCS

While the previous section provides a robust case in favour of large-scale CCS, we have also outlined the strength of some moral concerns surrounding such an ambition. In this section, we build our case that, if done the right way, pursuing the former can in fact alleviate the latter.

We adopt the view put forward by Lane et al. (2021) that the uncertainties associated with practicable storage prospects will likely be the limiting constraint on how fast the overall CCS industry can grow.^6^ Lane et al. (2021) outline that, for any region to maximize its potential for storage development, two crucial requirements are: (1) strategic up-front investments that improve understanding of decadal prospects for sustained CO_2_ injection; and (2) sufficient local institutional capability to effectively understand and manage the technical and commercial risks associated with that judgement. We call plans to deliberately address those two goals the *strategic pursuit* of CCS.

The implications of this somewhat novel^7^ perspective are fourfold. The first three are practical in nature; the third and fourth also address the moral motivations and objections relating to CCS.

First, the planning mindset needs to shift from assuming that a strategic commitment *will* stimulate actual CCS activity to one that accepts the need for strategic funding to ascertain *whether* substantive local CCS growth is plausible at all.

Second, the strategic commitment required is substantial, because if we are to achieve anywhere near the rates of CCS industry growth implied in the deep decarbonisation scenarios compliant with the PA target, the need for early stimulus funding is orders of magnitude greater than what is currently on offer in any country.^8^

The third implication is crucial, because it illustrates the urgency of those strategic investments. Following such an investment, planners could expect timelags of a decade or more before meaningful rates of CO_2_ storage development would be realised. At that point, the sheer scale and complexity of the necessary infrastructure build-out^9^—akin to creating all the world’s oil and gas infrastructure in a few short decades—suggests it unlikely that future policy could make up for time lost through short term delays. Following the rationale provided above, any such delays would therefore serve to reduce the overall mitigation contribution that CCS could make. Initiating strategic investments into CO_2_ storage assessment, in spite of the risks involved, is therefore a prudentially necessary means to delivering on the moral case in favour of pursuing CCS.

Fourth, the act of delivering on a strategic pursuit of CCS will address and alleviate the strongest moral objections identified above (§2.1). Investments into the appraisal of practicable storage opportunity will both reduce uncertainty on injection rates that can be sustained over the long term, and incorporate detailed analysis of geological, environmental and social impacts associated with local CO_2_ injection activities. That information can’t fully eliminate the possibility of moral hazard and hubris corrupting the decarbonisation planning process, but it does help to curb inflated expectations. Furthermore, emphasising the uncertainty of the ultimate storage opportunity to decision-makers could reduce moral hazard (Grant et al., 2021); if we do not know what the actual geological options for CO_2_ storage are—and it is a real possibility that they are insufficient—it is harder to rely on CDR-CCS as backstopping insufficient mitigation.

In summary, a strategic commitment to developing CO_2_ storage opportunity both is a necessary prerequisite to deliver on the moral arguments for pursuing CCS and will reduce the expectational and operational risks. By maximising the moral pros, and minimising the moral cons, it can greatly weaken the oversimplified options that have bedevilled interest in CCS. Given that, we endorse the strategic *pursuit* of global-scale CCS as a morally justified ambition. However, as we outline in the following section, the question of how best to make that pursuit is itself subject to morally conflicting considerations.

## Where to Direct Funding for Carbon Capture and Storage

Having established the claim that it is morally justified to pursue CCS through strategic investment into assessing CO_2_ storage opportunity in the first half of the paper, we now address the question of *where* (regionally) that CCS stimulus funding should be directed. Our primary claim is that this question is subject to competing moral considerations that will be non-trivial to adjudicate.

First, we explain the moral considerations which support funding being directed towards developing Asian countries, appealing to immediate global distributive justice and the destabilisation that would result from not having access to CCS. Second, we explain the contrasting moral case, that the interests of those same countries are served by directing the available CCS stimulus funding towards wealthier regions with the best prospects for successful CCS deployment. Third, we discuss the trade-offs involved.

### The Case for Supporting CCS in Regions with the Greatest Need

We first explain one side of the tension by outlining two independent lines of reasoning to support the case for directing international funds to stimulate the pursuit of CCS in regions with the greatest expected needs in the coming decades, predominantly developing Asian countries.

Both lines of reasoning build on the notion that the need for FF-CCS is particularly strong in *developing Asia*, home to the world’s largest fleet of steel, cement, fertiliser, chemicals, and coal-fired power plants, the majority not reaching their economically optimal retirement age for some decades. Those stakeholder groups represent some of the most difficult mitigation challenges in the global industrial system, with FF-CCS viewed as crucial for domestic decarbonisation, because of the extremely constrained set of alternatives at their disposal (IEA, 2019b).

While the motivation for having FF-CCS as an option for developing Asia is strong, the motivation for those countries to commit the necessary strategic investments seems low. They lack the informational and institutional capabilities needed to expedite CO_2_ storage infrastructure development, and we observe a historical paucity of developing country interest in dedicated CO_2_ storage investigations (Lane et al., 2021). The prospects of that changing seem bleak; investments into dedicated CO_2_ storage investigations carry a material risk that they will deliver little to no mitigation value, hence will struggle for priority in countries wishing to direct their scarce resources to more immediate priorities such as poverty alleviation.

In the absence of sufficient domestic motivation (cf. Roman, 2011), international financial support for CO_2_ storage appraisal could activate the steps needed for a strategic pursuit of CCS in developing Asia. We offer two moral considerations that support such a flow of funding.

First, decarbonising developing Asia without recourse to FF-CCS could create substantial community-level and macro-economic disruption if it leads to the premature closure of those industries or imposes a substantial barrier to pursuing future economic growth stimulated by industrialisation. Such outcomes could violate both the Rawlsian need to respect citizens’ legitimate expectations for development (Meyer & Sanklecha, 2014) and Moellendorf’s (2014) requirement that developing country mitigation not imperil their development. Furthermore, Moellendorf’s Anti-Poverty Principle also provides a more direct justification for the direction of international finance to support CCS development, in order to keep developing country mitigation costs to a minimum.

In the absence of stable international settings to align development and decarbonisation decision making, international financial support for CO_2_ storage appraisal offers a pathway to bridge the two objectives. Therefore, with CCS established as a morally Justifiable Regional Solution, and the imperative of urgency described above, we establish our first line of reasoning that there is a (defeasible) moral obligation to provide international finance support.

Second, directing international finance to stimulate the pursuit of CCS in developing Asia would also promote global distributive justice. This is a morally robust consideration, in the sense that it applies regardless of the specific shape or pattern of global justice one favours. For instance, the flow of international funding to developing Asia is likely to contribute to greater overall welfare (utilitarianism), greater distributionally-sensitive weighted welfare (prioritarianism), more people being raised to a sufficient level of resources (sufficientarianism) and more globally equitable distribution of resources (egalitarianism).

### The Case for Supporting CCS in Regions with the Best Prospects

For our contrary argument, we again appeal to two independent moral considerations. Both justify a priority towards CCS efforts in regions that have the best prospects for high-rate CO_2_ sequestration over the long term.

Berly and Garnett (2018) advocate that the way to maximise the overall rate of CCS deployment is to prioritise storage development in locations with the best prospects for sustained high-rate CO_2_ injection. They identify that relatively few *regions with the best prospects* have (a) the lowest risk for short term storage prospects, and (b) confidence in the potential for a very large-scale future expansion of CO_2_ injection in that same location.^10^ It can also be inferred that the CO_2_ sequestration payoff from a unit investment into storage investigations will (likely) increase super-linearly, the better the site matches those Berly and Garnett criteria.

That approach to considering regions with best prospects can also be applied at the international level. While Lane et al. (2021) identify a lack of useful research on regionally-varying storage prospects, their analysis indicates that the likelihood of achieving the levels of regional CCS activity implied in PA-compliant scenarios is much lower for Southeast Asia, China and (particularly) India, than it is for the Europe, the Middle East, Russia and the USA. Those differences recognise both the relatively poor geological prospects in developing Asian countries, but also a lack of CCS-relevant institutional capabilities. Given that, we posit that (1) the relatively poor geological and institutional capabilities of developing Asia imply a greater risk that strategic investments into a pursuit of CCS will fail; and (2) even if those investments don’t fail, it’s likely they will deliver a far lower mitigation return-on-investment than if spent in a country with better storage prospects.

In light of those practical concerns, we identify two moral considerations for supporting CCS investigations in regions with the best geo-storage prospects.

First, a utilitarian case can be made that CCS stimulus funding should be directed where it delivers the greatest overall mitigation benefit, on the grounds that cost-effective mitigation options are superior to lesser alternatives. Since the development of practicable storage opportunity will be the mitigation limiting constraint for CCS, there is the general possibility that utilitarian considerations will support targeting financing towards regions with the best storage prospects. In contrast, it is likely that strategic CCS investments into developing Asia provide a relatively poor value proposition.

This is the first moral tension. A decision to direct international financial support to stimulate the pursuit of CCS in developing Asia, while compatible with the *Justifiable Regional Solution* argument, would likely deliver less of the global public good that motivates the *Necessary Global Solution* view of CCS.^11^

A second relevant consideration is the heterogeneous distribution of impacts caused by climate change, expected to fall disproportionately on those in the Global South—and developing Asia in particular. In other words, a failure to deliver on CO_2_ mitigation objectives will exacerbate long-term global inequalities.

These independent moral considerations suggest that a case can be made for directing CCS funding towards the regions with best prospects. In the following section, we lay out different axes along which these considerations are in tension with the considerations for directing funding to developing Asia.

### A Morally Non-Trivial Choice

The Justified Regional Solution motivation suggests that we should prioritise regions which have the most expected need for CCS, which broadly speaking picks out developing Asian countries. In doing so, we would also advance global distributive justice considerations, at least for the short term. The Necessary Global Solution argument suggests that we should maximise our carbon dioxide removal capacity, which suggests that our funding should target regions with the best expected long-term storage potential.

We see two trade-offs arising in this context. The first is global total versus regional needs-based use of CCS. Thinking globally would support maximising strategic CCS pursuit, and funding countries with best storage CCS prospects in expectation, countries which tend to be wealthier. Thinking regionally would support expanding CCS capacities in countries which will have CCS needs in the coming years.

The second is long-term versus short-term distributive justice. In the long-term, using the same resources to maximise CCS potential in expectation maximises the carbon dioxide removal benefits, which are long-term global public goods. In the short-term, directing CCS funding towards globally unprivileged countries will help address significant extant distributive inequalities.

We conclude that this choice is morally non-trivial and sensitive to which values we want to adopt.

## Conclusions

The question of whether pursuing CCS strategically represents the best use of resources all things considered is still open. Encouraging behavioural change and increasing less radical forms of mitigation are important and required. However, the Necessary Global Solutions and Justifiable Regional Solution Arguments suggest that, given the sheer scale of CCS estimated to be needed to reach the PA target, pursuing CCS should be a (significant) part of the portfolio of climate responses. By drawing attention to the lead times and the importance of stimulating CCS storage capacity in a strategic way, we indicate that the preparation has to be done carefully and early to have any chance for success and to help address the moral challenges that have been raised to CCS.

If we are right that storage capacity will act as a bottleneck on the ability for CCS to contribute in the coming decades, then that generates other difficult questions we were unable to address here. For instance, how should FF-CCS and CDR-CCS trade against each other if competing for the same geological storage capacity? How will earlier as opposed to later investment affect the overall storage capacity? Will carbon utilisation opportunities help address geological storage limitations? Can transport of CO_2_ decouple the storage and capture processes of CCS, allowing us to creative ways of addressing the regional dilemma we developed herein?

However, our view is that thinking about the rate of injection into storage capacity focuses the moral questions about CCS in helpful ways. After all, while the pressing temporal nature of *when* to develop CCS is well-recognised, the regional question of *where* demands novel research with practical understanding.

## Footnotes

1 We use the FF-CCS terminology to also include CO_2_ produced from chemical conversion of non-fossil feedstocks in industrial cement production.

2 Our differentiation between FF-CCS and CDR-CCS aligns quite neatly with Heyward’s (2013) distinction between mitigation and CDR. CDR-CCS approaches are (1) growing biomass for energy combustion, then capturing the CO_2_ for geological sequestration, often referred to as bioenergy plus CCS (BECCS); and (2) direct capture of CO_2_ from air using engineering technologies for subsequent geological sequestration (DACCS).

3 Preston (2013) provides a clear overview of, and a useful framework for distinguishing between, the spectrum of moral concerns associated with geoengineering and CDR, some of which are relevant for considering CCS specifically.

4 Taebi (2017) adds that social acceptability need neither entail, nor be entailed by, ethical acceptability. We agree with Taebi that social acceptability is a component of a broader and more complex matrix of moral considerations.

5 While we lack sufficient space here, in a companion piece called “How Much Carbon Capture and Storage to Pursue” (in preparation), we explain the importance of a strategy that involves *both* FF-CCS and CDR-CCS. There are two related reasons for this. First, since storage can be used for both fossil fuel and CDR purposes, if a strategy only considers one of these, then the storage developed may be insufficient to meet the combined needs, generating conflict for storage. Second, if we limit storage to (expected) CDR needs, we have few ways to address hard to decarbonize sectors (such as steel and cement). Failure to store emissions from these sectors now exacerbates subsequent demand for storage, undermining the CDR potential for storage.

6 Those uncertainties are highly localized, inherent to even the best prospective locations for geological storage, and costly assessments of practicable storage opportunity will always carry the risk that the investment yields nothing useful.

7 We use the term ‘somewhat novel’ carefully. Lane et al. (2021) illustrate that the key principles at play are deeply rooted in operational expertise, and have long been visible in the academic literature. However, it appears they have not been widely debated nor broadly recognised across much of the broader CCS research community.

8 The USA, being one of the most advanced countries in terms of CCS development, provides an ample illustration of that point. Despite having hundreds of million dollars over 20 years to better understand storage geology, Larson et al (2020) suggest that US$1.5 billion is required for strategic storage appraisal in the next 5 years, followed by a further $11.5 billion to ascertain injection rates that are commercially viable and socially acceptable. That would strive for a mid-century CCS target of ~ 1 Gt of CO_2_ sequestered per year, which is of similar order to the PA-compliant mid-century scenarios for each of the US, EU, China and India.

9 The staggering rate of project development required—30–60 new projects every year for multiple decades (IEAGHG, 2017)—is not the only challenge. Zapantis et al. (2019) also outlines the critical importance of government investment into building shared CO_2_ transport and storage infrastructure to reduce the counter-party commercial risk for private investors.

10 This represents a recent shift in the CCS research community’s rhetoric on how best to reach the global-scale CCS targets implied in PA-compliant scenarios. Historically, a more common view (e.g. Reiner, 2016) was that widespread stimulus of CCS in as many regions as possible is crucial to expediting global deployment.

11 Our use of the term “global public good” indicates that the benefits are nonrival and nonexcludable, not that the overall effects on all agents are net positive [cf. the discussion between Gardiner (2013, 2014) and Morrow (2014)].
